# Future CO_2_-induced seawater acidification mediates the physiological performance of a green alga *Ulva linza* in different photoperiods

**DOI:** 10.7717/peerj.7048

**Published:** 2019-06-04

**Authors:** Furong Yue, Guang Gao, Jing Ma, Hailong Wu, Xinshu Li, Juntian Xu

**Affiliations:** 1 Jiangsu Key Laboratory of Marine Bioresources and Environment, Huaihai Institute of Technology, Lianyungang, China; 2 Co-Innovation Center of Jiangsu Marine Bio-industry Technology, HuaiHai Institute of Technology, Lianyungang, China; 3 Jiangsu Key Laboratory of Marine Biotechnology, Huaihai Institute of Technology, Lianyungang, China

**Keywords:** Growth, Ocean acidification, Photoperiod, Photosynthesis, *Ulva linza*

## Abstract

Photoperiods have an important impact on macroalgae living in the intertidal zone. Ocean acidification also influences the physiology of macroalgae. However, little is known about the interaction between ocean acidification and photoperiod on macroalgae. In this study, a green alga *Ulva linza* was cultured under three different photoperiods (L: D = 8:16, 12:12, 16:8) and two different CO_2_ levels (LC, 400 ppm; HC, 1,000 ppm) to investigate their responses. The results showed that relative growth rate of *U. linza* increased with extended light periods under LC but decreased at HC when exposed to the longest light period of 16 h compared to 12 h. Higher CO_2_ levels enhanced the relative growth rate at a L: D of 8:16, had no effect at 12:12 but reduced RGR at 16:8. At LC, the L: D of 16:8 significantly stimulated maximum quantum yield (Yield). Higher CO_2_ levels enhanced Yield at L: D of 12:12 and 8:16, had negative effect at 16:8. Non-photochemical quenching (NPQ) increased with increasing light period. High CO_2_ levels did not affect respiration rate during shorter light periods but enhanced it at a light period of 16 h. Longer light periods had negative effects on Chl *a* and Chl *b* content, and high CO_2_ level also inhibited the synthesis of these pigments. Our data demonstrate the interactive effects of CO_2_ and photoperiod on the physiological characteristics of the green tide macroalga *Ulva linza* and indicate that future ocean acidification may hinder the stimulatory effect of long light periods on growth of *Ulva* species.

## Introduction

Due to the activities of humans, the concentration of atmospheric CO_2_ has increased to 400 ppm from 278 ppm during the pre-industrial revolution (Gattuso et al., 2015). It is estimated that the oceans, as a CO_2_ sink, have taken up approximately 48% of the fossil-fuel and cement-manufacturing emissions (Sabine et al., 2004). The concentration of atmospheric CO_2_ has been predicted to reach almost 1,000 ppm by the end of 21st century (Gattuso et al., 2015). This would result in the decline of surface ocean pH by 0.3–0.4 units, an increase of hydrogen ion concentration and bicarbonate concentration in seawater, which is termed ocean acidification (OA) (Orr et al., 2005; Koch et al., 2013; Albright, 2016). OA will be maintained for hundreds of years, and thus could cause a huge change in marine ecosystems (Kroeker et al., 2013; Wittmann & Pörtner, 2013).

Marine phytoplankton accounts for 50% of global primary production (Beardall, Stojkovic & Larsen, 2009) and macroalgae accounts for 10% of marine primary productivity (Smith, 1981; Charpy-Roubaud & Sournia, 1990; Heinrich et al., 2012). Macroalgae have evolved distinct strategies to capture inorganic carbon (Ci) for photosynthesis: (1) by the diffusive uptake of CO_2_ (Giordano, Beardall & Raven, 2005; Raven et al., 2005), (2) the active transport of HCO${}_{3}^{-}$/CO_2_ (termed carbon concentrating mechanisms (CCMs) (Maberly, Raven & Johnston, 1992; Raven, 2003; Giordano, Beardall & Raven, 2005). The elevated CO_2_ might enhance the growth and photosynthesis of algae that depend on CO_2_ diffusion or have weak CCMs (Kübler, Johnston & Raven, 1999; Gao et al., 2016a). Therefore, the physiological influences of OA on macroalgae might be species-specific. For example, OA has a positive influences on the growth of macroalgae, such as the red algae *Pyropia yezoensis, Pyropia haitanensis* and *Gracilaria lemaneiformis* (Gao et al., 1991; Xu & Gao, 2015; Chen et al., 2018), green algae *Ulva prolifera*, *Ulva lactuca, Caulerpa taxifolia* (Xu & Gao, 2012; Chen, Zou & Jiang, 2015; Roth-Schulze et al., 2018) and brown algae *Hizikia fusiforme*, *Ecklonia radiata*, *Sargassum muticum* (Zou, 2005; Britton et al., 2016; Xu et al., 2017). However, research has shown that high CO_2_ levels did not have a detectable effect on the relative growth rate of several species, for example, *Sargassum henslowianum*, *Ulva rigida*, *Ulva australis*, the giant kelp *Macrocystis pyrifera* and *Ulva australis* (Chen & Zou, 2014; Rautenberger et al., 2015; Fernández, Roleda & Hurd, 2015; Reidenbach et al., 2017). Furthermore, increasing seawater CO_2_ concentration reduced the growth of *Gracilaria tenuistipitata,* ***Porphyra leucosticte***, *Fucus vesiculosus*, *Halimeda opuntia* and *Ulva linza* (García-Sánchez, Fernández & Niell, 1994; Mercado et al., 1999; Gutow et al., 2014; Johnson, Price & Smith, 2014; Gao et al., 2018). In addition, the various effects of ocean acidification may be also modulated by other environmental factors, such as temperature, nutrients and photoperiods (Gao et al., 2017a; Gao et al., 2019; Li et al., 2018).

Photoperiod is a key factor regulating seasonal responses of algae (Dring, 1988). Furthermore, daylength can influence algal affinity for dissolved inorganic carbon and cellular carbon demand, thus imposing an effect on CCMs regulation (Rost, Riebesell & Sültemeyer, 2006). Thus, the photoperiod may be critical for algae cultured under elevated CO_2_ levels. Meanwhile, the interactive effects of OA and increased photoperiods could enhance the growth of *U. prolifera*, thus increase the opportunities for occurrence of green tides (Li et al., 2018). Elevated CO_2_ levels enhanced the photosynthetic rate, but longer illumination periods reduced the photosynthetic efficiency in *E. huxleyi* (Nielsen, 1997; Rost, Riebesell & Sültemeyer, 2006). Therefore, at HC, the responses of algae to different photoperiods appear to be genera- and species-specific.

Green macroalgal bloom has occurred each year in the Yellow Sea (YS) since 2007, termed green tides (Xu, Zhang & Cheng, 2016). Green tides generate aesthetic problems and toxic gas when thalli decompose (Gao et al., 2016b). *Ulva* is the dominant genus contributing majority of green tides and *U. linza* is one of species causing green tides in the Yellow Sea (Fletcher, 1996; Kang et al., 2014; Gao et al., 2017a; Gao et al., 2017b). However, little is known about the interactive effects of ocean acidification and photoperiod on *U. linza*. Previous study showed that the effect of ocean acidification on diatoms was related to light intensity (Gao et al., 2012). In this study, based on previous studies, we hypothesized the effect of ocean acidification on *U. linza* may dependent on photoperiod. To test our hypothesis, the physiological responses of the green macroalga *Ulva linza* under two different CO_2_ levels and three different photoperiods were examined.

## Materials & Methods

### Thalli collection and culture conditions

*U. linza* was collected from the coastal water of Gaogong peninsula (119.3°E, 34.5°N), Lianyungang, Jiangsu Province, China. Gaogong peninsula is a public place and no approval is required for collecting naturally growing *Ulva* species in China because *Ulva* species cause green tides in coastal waters of the Yellow Sea. *U. linza* was identified by morphological characters (Ma et al., 2009). The samples were transferred to the laboratory in a portable cooler (4–6 °C) box within one hour. Healthy thalli (first observed by color and then checked with maximum quantum yield of PSII) were selected and cleaned with filtered natural seawater to remove sediments, visible epiphytes and attached animals. Thalli were pre-cultured in a 500 ml flask in an illuminated incubator (GXZ-500B, Ningbo, China) at 20 °C, with the illumination intensity set at 150 µmol photons m^−2^s^−1^ (12L:12D). Sterilized filtered seawater (salinity 30, supplied with 8 µM NaH_2_PO_4_ and 60 µM NaNO_3_) was used as culture medium and the medium was bubbled with air before being renewed every two days.

For these experiments, approximately three 1 cm long thalli were placed into a 550 ml flask filled with 500 ml sterile seawater aerated ambient outdoor air (400 ppm, LC) or with CO_2_-enriched air (1,000 ppm, HC) and randomly cultured in three separate incubators with different light levels (8L:16D, 12L:12D, 16L:8D) and continuous aeration at 20 °C with 150 µmol photons m^−2^s^−1^. Different CO_2_ levels (400 ppm, LC; 1,000 ppm, HC) were achieved by bubbling ambient air or CO_2_-enriched air via a CO_2_ plant incubator (HP 1000 G-D, Ruihua Instruments, Wuhan, China).

To maintain the pH_NBS_ at about 8.12 (LC) and 7.78 (HC) under different photoperiods, the increased algal biomass were removed constantly and the medium was changed every two days and daily variations in pH were maintained at less than 0.05. The experiment lasted 9 days, the physiological factors were taken on the last 3 days.

### Estimate of carbonate system parameters

The seawater pH was monitored with a pH meter (pH 700, Eutech Instruments, Singapore) and total alkalinity (TA) was calculated by titrations (Gao et al., 2019). Other parameters of the carbonate system were obtained with CO2SYS software (Pierrot, Lewis & Wallace, 2006), the equilibrium constants K_1_ and K_2_ for carbonic acid dissociation (Roy et al., 1993).

### Measurement of growth

The length of *U. linza* was recorded every 2 days. The relative growth rate (RGR) of thalli were calculated across the 6-day period before other physiological parameters were measured during the following 3 days. RGR was calculated as follows: RGR = ln (W_t_/W_0_)/t, where W_t_ is the length after t days culture, W_0_ is the initial length.

### Chlorophyll fluorescence measurements

Chlorophyll *a* fluorescence in *U. linza* was measured with a portable PAM (Pulse-amplitude-modulation; AquaPen-P AP-P 100, Chech). Algae were dark adapted for 15 min before the experiment. The parameters were calculated according to the following equations:

rETR = PAR × Y(II) × 0.84 × 0.5 (Schreiber, 2004; Zhang, Zhang & Yang, 2017),

where rETR is the relative electron transport rate; PAR is the actinic light; Y(II) is the effective quantum yield of PSII.

NPQ = Fm − Fm’/1 (Bilger & Schreiber, 1986),

where NPQ is the non-photochemical quenching; Fm is the maximum fluorescence value of *U. linza* when they were adapted in the dark for 15 min; Fm’ is the maximum fluorescence value of *U. linza* under actinic light conditions.

### Respiration rate measurements

The respiration rate of *U. linza* was measured using a Clark-type oxygen electrode (YSI Model 5300, Yellow Springs Instrument Co., USA). The thallus was cut into 1 cm long segments with scissors and the thalli were placed in culture conditions for at least 1 h to decrease the effects of cutting damage. Approximately 0.01 g fresh weight of thalli were placed in the reaction chamber with 8 ml medium. Temperature was controlled at 20 °C with a circulating water bath. The decrease of the oxygen content in the seawater in darkness with seven minutes was defined as the respiration rate.

### Measurement of photosynthetic pigments

Chlorophyll *a* and *b* were extracted from thalli (about 10 mg FW) with 5 ml methanol at 4 °C for 24 h in the dark. The absorption values were obtained at 652 nm, 663 nm and 665 nm using an ultraviolet spectrophotometer (Ultrospect 3300 pro; Amersham Bioscience, Sweden). The contents of the Chl *a* and Chl *b* were estimated using the method of Porra, Thompson & Kriedemann (1989).

### Data analysis

All the data are shown as mean ± SD. Origin 9.0 and SPSS 18.0 were used to analyze data. Two-way ANOVA was used to assess the interactive effects of CO_2_ levels and photoperiods on relative growth rate, chlorophyll fluorescence parameters, respiration rate and pigment content of *U. linza*. One-way ANOVA was used to analyze differences under the same conditions. Tukey HSD was conducted for *post hoc* investigation. Confidence intervals were set at 95%.

## Results

Both the elevated CO_2_ levels and photoperiod altered carbonate parameters in seawater, and they both had an interactive effect (Tables 1 and 2). The elevated CO_2_ decreased pH and CO${}_{3}^{2-}$, increased *p* CO_2_, DIC, HCO${}_{3}^{-}$ and CO_2_ in the seawater. Increased photoperiod did not affect carbonate parameters at LC but the longest photoperiod increased DIC, HCO${}_{3}^{-}$ and TA compared to shortest photoperiod.

**Table 1:** Parameters of the seawater carbonate system in different cultures.

	pH_NBS_	*p* CO_2_ (µatm)	DIC (µmol kg^−1^)	HCO${}_{3}^{-}$ (µmol kg^−1^)	CO${}_{3}^{2-}$ (µmol kg^−1^)	CO_2_ (µmol kg^−1^)	TA (µmol kg^−1^)
8:16 LC	8.14 ± 0.02^a^	580.32 ± 35.74^a^	2597.04 ± 57^a^	2385.19 ± 56.15^a^	192.59 ± 7.23^a^	19.56 ± 1.19^a^	2850.78 ± 56.26^a^
12:12 LC	8.12 ± 0.01^a^	613.13 ± 11.21^a^	2594.32 ± 61.96^a^	2391.03 ± 55.85^a^	182.94 ± 6.07^a^	20.35 ± 0.37^a^	2834.31 ± 67.99^a^
16:8 LC	8.13 ± 0.01^a^	608.54 ± 14.83^a^	2638.26 ± 40.24^a^	2427.94 ± 35.09^a^	190.12 ± 6.73^a^	20.19 ± 0.49^a^	2886.86 ± 46.67^a^
8:16 HC	7.74 ± 0.03^b^	1614.77 ± 100.05^b^	2780.28 ± 12.05^b^	2641.71 ± 12.23^b^	84.99 ± 5.08^b^	53.58 ± 3.32^b^	2854.16 ± 13.32^a^
12:12 HC	7.79 ± 0.02^c^^d^	1445.67 ± 58.75^c^	2799.47 ± 14.61^b^	2655.70 ± 13.74^b^	95.80 ± 3.81^b^	47.97 ± 1.95^c^	2893.15 ± 17.18^a^
16:8 HC	7.75 ± 0.01^b^^d^	1652.45 ± 43.38^b^	2938.18 ± 47.63^c^	2790.86 ± 45.52^c^	92.48 ± 1.28^b^	54.83 ± 1.44^b^	3019.14 ± 46.95^b^

Data are the mean ± SD (*n* = 3).

DIC dissolved inorganic carbon TA total alkalinity

**Table 2:** Two-way ANOVA analysis of variance for the effects of CO_2_ and photoperiod regimes on pH, *p* CO_2_, dissolved inorganic carbon (DIC), HCO${}_{3}^{-}$, CO${}_{3}^{2-}$, CO_2_ and total alkalinity (TA) in seawater.

Source		pH		*p* CO_2_		DIC		HCO${}_{3}^{-}$		CO${}_{3}^{2-}$		CO_2_		TA	
	df	F	Sig.	F	Sig.	F	Sig.	F	Sig.	F	Sig.	F	Sig.	F	Sig.
Photoperiod	2	1.333	0.3	5.640	0.019	9.697	0.003	10.112	0.003	.353	0.710	5.632	0.019	8.638	0.005
CO_2_	1	2268.750	<0.001	1498.294	<0.001	125.390	<0.001	236.530	<0.001	1453.669	<0.001	1497.372	<0.001	8.977	0.011
Photoperiod*CO_2_	2	7.750	0.007	7.562	0.007	3.054	0.085	3.184	0.078	5.342	0.022	7.557	0.008	2.976	0.089
Error	12														

**Notes.**

CO_2_*photoperiod means the interactive effects of CO_2_ and photoperiod, df mean degree of freedom and F means the value of F statistic, and Sig. means *p*-value.

**Figure 1:**
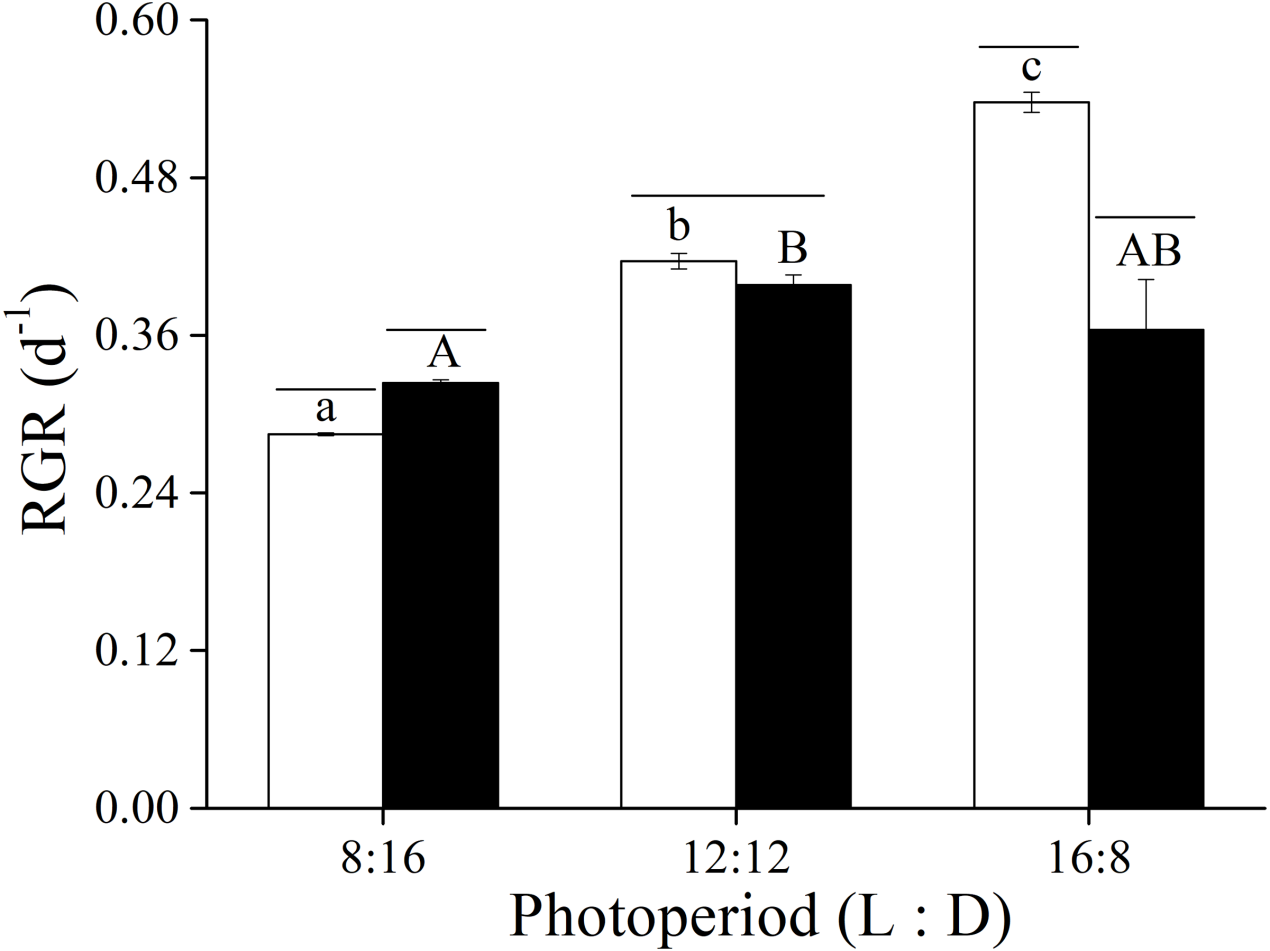
Relative growth rate. Relative growth rate (RGR) of *U. linza* thalli grown at different CO_2_ levels (400 ppm, LC; 1000 ppm, HC) and different photoperiods (L: D = 8:16, 12:12, 16:8).

The two-way ANOVA showed that elevated CO_2_ and photoperiod had an interactive effect, and both elevated CO_2_ levels and the photoperiods had a significant effect on the RGR of *U. linza* (Fig. 1 and Table 3). At LC, the RGR of adult *U. linza* increased with the extended light periods, and the highest RGR occurred at a L: D of 16:8. The effect of CO_2_ also varied with photoperiod. Higher CO_2_ levels enhanced RGR at L: D of 8:16, but had no effect at 12:12 and reduced it at 16:8.

**Figure 2:**
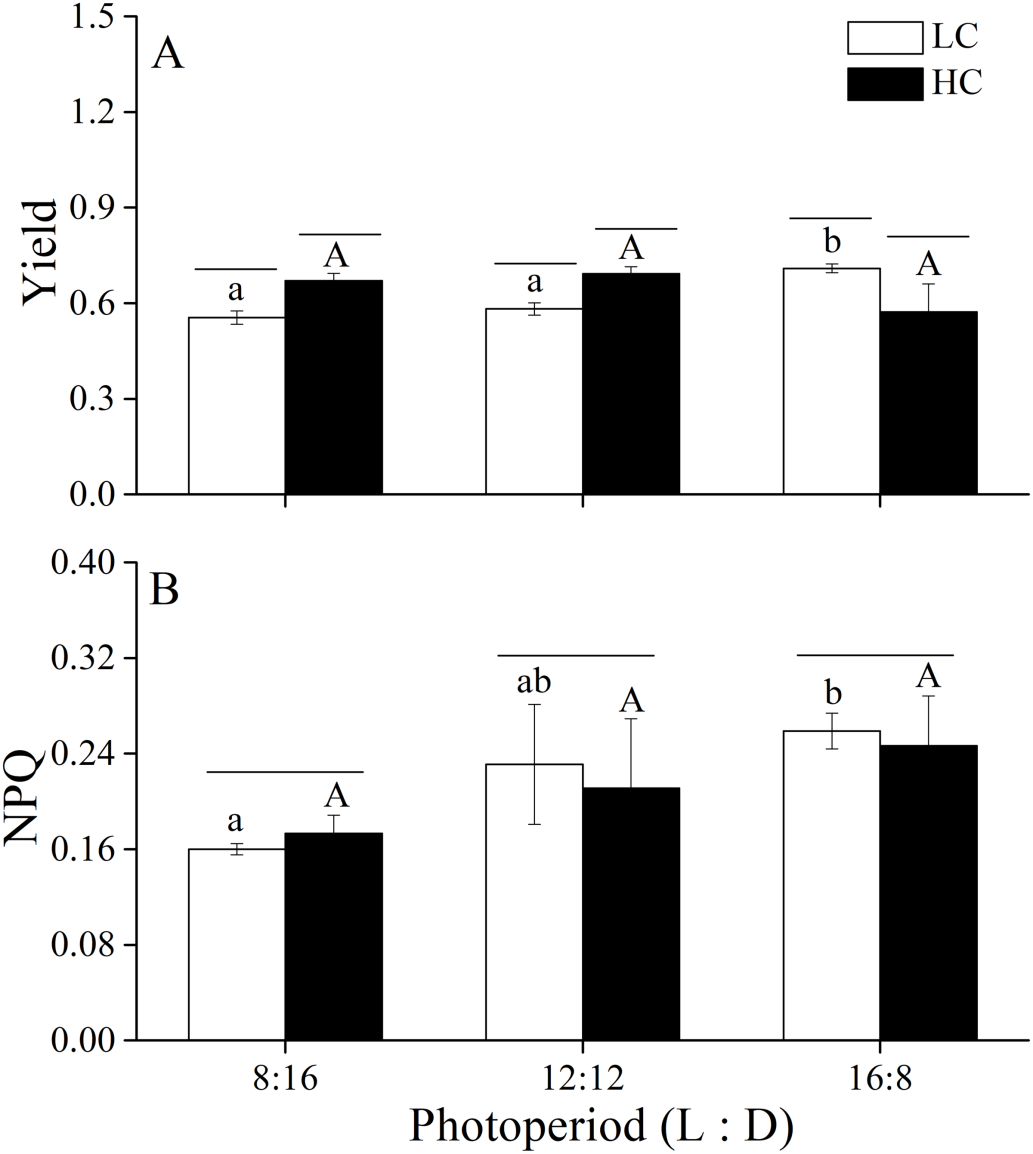
Yield and NPQ. Yield (A) and NPQ (B) of *U. linza* thalli grown at different CO_2_ levels (400 ppm, LC; 1000 ppm, HC) and different photoperiods (L: D = 8:16, 12:12, 16:8).

The Yield and NPQ were measured under different CO_2_ levels and photoperiod conditions (Fig. 2). Two-way ANOVA showed that elevated CO_2_ and photoperiod had an interactive effect on Yield (Table 4). Higher CO_2_ levels increased Yield when thalli were cultured under photoperiods of 8:16 and 12:12 but reduced it under 16:8. Photoperiod had the main effect on NPQ (Table 4). At LC, thalli cultured at L: D of 16:8 had higher NPQ compared to L: D of 8:16 while the difference between 8:16 and 12:12 was insignificant. At HC, NPQ increased with the increase in photoperiod although the increase was not statistically significant. The elevated CO_2_ had neutral effect on NPQ of *U. linza*.

**Table 3:** Two-way ANOVA analysis of variance for the effects of CO_2_ and photoperiod regimes on RGR of *Ulva linza* cultured at different CO_2_ levels and photoperiod regimes.

Source	df	F	Sig.
Photoperiod	2	124.249	<0.001
CO_2_	1	42.292	<0.001
Photoperiod* CO_2_	2	65.959	<0.001
Error	12		

**Notes.**

CO_2_*photoperiod means the interactive effects of CO_2_ and photoperiod, df mean degree of freedom and F means the value of F statistic, and Sig. means *p*-value.

**Figure 3:**
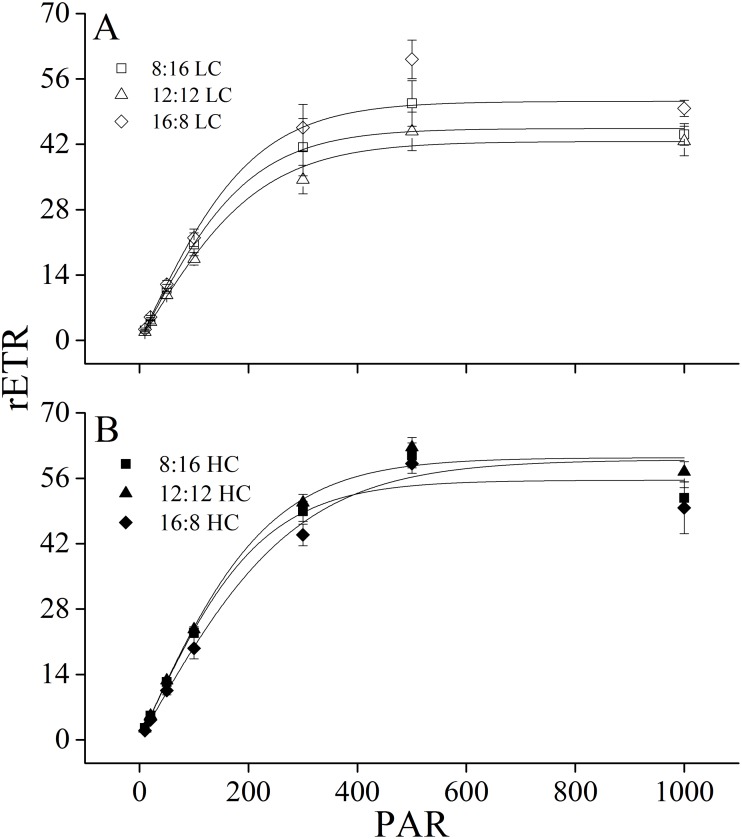
Rapid light curves. Rapid light curves (RLC) of *U. linza* thalli grown at different CO_2_ levels (400 ppm, LC; 1,000 ppm, HC) and different photoperiods (L: D = 8:16, 12:12, 16:8).

Maximum rETR (rETRmax), efficiency of electron transport (*α*), and saturating irradiance (I_k_) were calculated from the rapid light curves (Fig. 3, Table 5). Photoperiod and elevated CO_2_ levels had an interactive effect, and elevated CO_2_ levels had a main effect on light-saturated electron transport rate (rETRmax) (Table 6). Higher CO_2_ levels increased rETRmax during the 8:16 and 12:12 photoperiods, but did not affect it at 16:8. A similar pattern was also found for *α*. In contrast to rETRmax and *α*, CO_2_ did not affect I_k_ while photoperiod had the main effect on it. At LC, I_k_ increased when L: D rose from 8:16 to 12:12 but did not change with the further increase in photoperiod. At HC, I_k_ did not change when L: D rose from 8:16 to 12:12 but was enhanced when L: D increased to 16:8 (Table 5).

**Figure 4:**
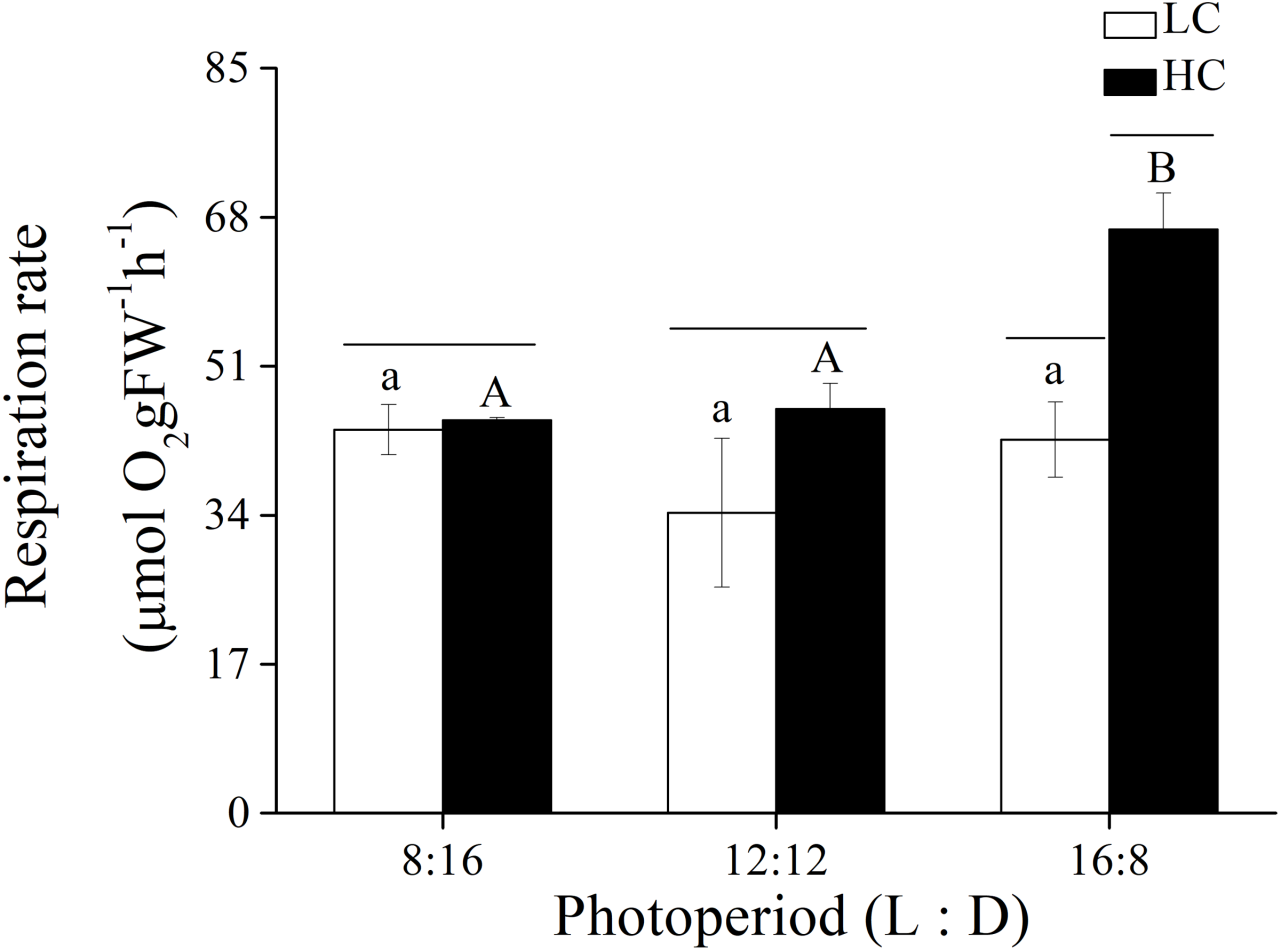
Respiration rate. Respiration rate of *U. linza* thalli grown at different CO_2_ levels (400 ppm, LC; 1,000 ppm, HC) and different photoperiods (L: D = 8:16, 12:12, 16:8).

In addition to photosynthetic parameters, the effects of *p* CO_2_ and photoperiod on the respiration rate of adult *U. linza* were also investigated (Fig. 4). Photoperiod and elevated CO_2_ levels had an interactive effect, and both photoperiod and elevated CO_2_ levels had the primary effect on the respiration rate of *U. linza* (Table 7). Higher CO_2_ levels did not affect the respiration rate at photoperiods of 8:16 or 12:12 but increased it by 56.39% at a photoperiod of 16:8.

**Figure 5:**
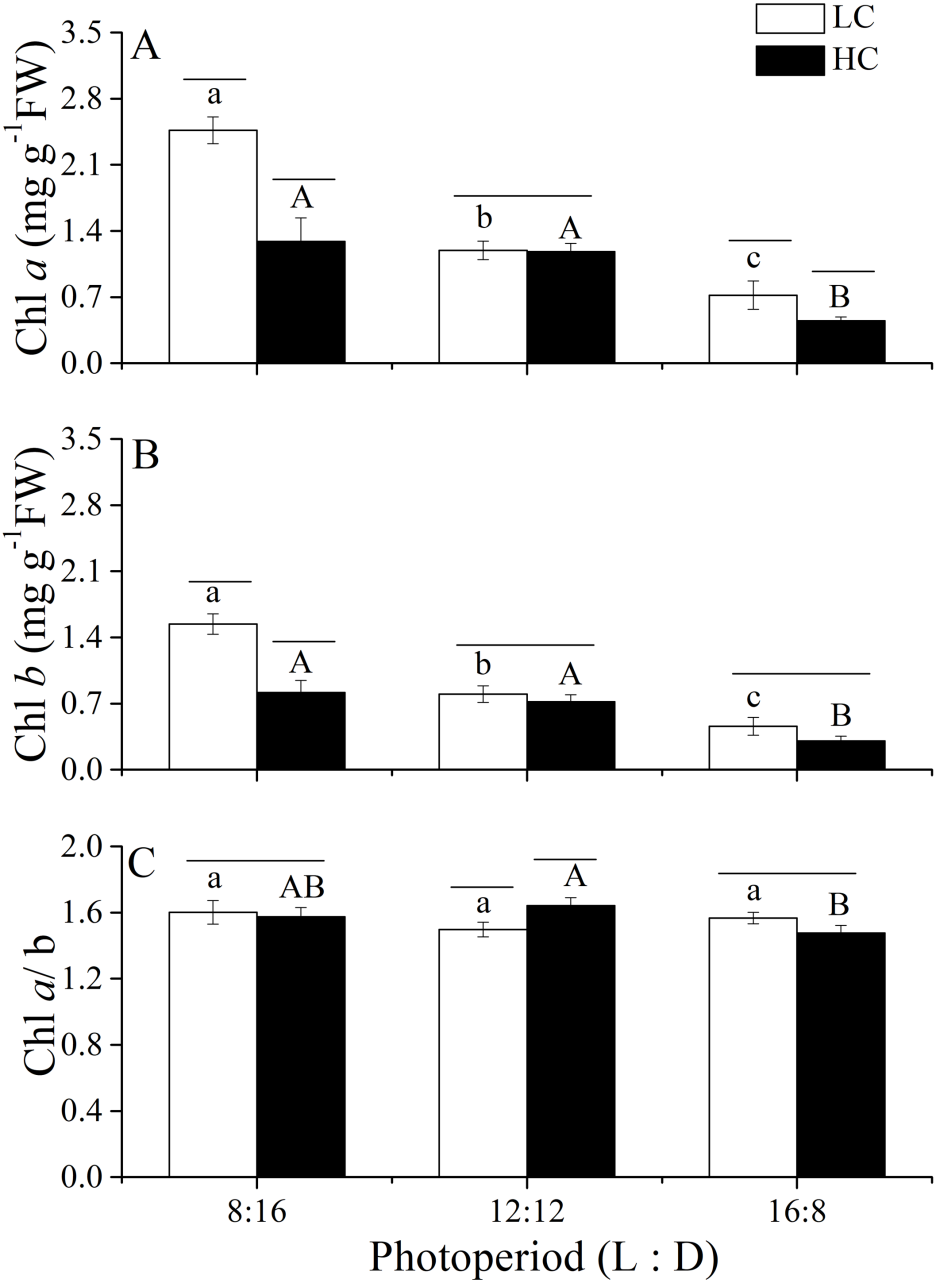
Pigment contents and the Chl *a.*/*b* ratio. Pigment contents (Chl *a*, (A); Chl *b*, (B)) and the Chl *a*/*b* ratio (C) of *U. linza* thalli grown at different CO_2_ levels (400 ppm, LC; 1,000 ppm, HC) and different photoperiods (L: D = 8:16, 12:12, 16:8).

Changes in photosynthetic pigments of *U. linza* grown under various conditions are shown in Fig. 5. Two-way ANOVA showed that CO_2_ and photoperiod had an interactive effect, and both CO_2_ levels and the photoperiods had the main effect on the Chl *a* content of *U. linza* (Table 8). Prolonged light periods reduced the synthesis of Chl *a* in thalli although the difference between the photoperiods of 8:16 and 12:12 at HC was not statistically significant. Higher CO_2_ levels reduced Chl *a* at the photoperiods of 8:16 and 12:12 but did not affect it at a L: D of 12:12. The same trend was found for Chl *b*. The Chl *a*/*b* ratios were all greater than 1.0, suggesting a higher synthesis of Chl *a* than Chl *b* under all culture conditions. Photoperiod and elevated CO_2_ levels had an interactive effect on the Chl *a*/*b* ratio (Table 8); the higher CO_2_ levels increased the ratio at a photoperiod of 12:12 but not at the other photoperiods.

**Table 4:** Two-way ANOVA analysis of variance for the effects of CO_2_ and photoperiod regimes on Yield and NPQ of *Ulva linza* cultured at different CO_2_ levels and photoperiod regimes.

Source	Yield			NPQ		
	df	F	Sig.	df	F	Sig.
Photoperiod	2	0.919	0.425	2	8.453	0.005
CO_2_	1	2.549	0.136	1	0.132	0.722
Photoperiod* CO_2_	2	19.786	<0.001	2	0.339	0.719
Error	12			12		

**Notes.**

CO_2_*photoperiod means the interactive effects of CO_2_ and photoperiod, df mean degree of freedom and F means the value of F statistic, and Sig. means *p*-value.

**Table 5:** Parameters of rapid light curves (RLC) of *U. linza* thalli cultured at different CO_2_ levels and photoperiod regimes.

	rETRmax	*α*	I_k_
8:16 LC	47.53 ± 3.63^a^	0.22 ± 0.04^a^	215.67 ± 19.89^a^
12:12 LC	43.87 ± 3.60^c^	0.18 ± 0.02^c^	248.87 ± 22.91^b^^d^
16:8 LC	54.83 ± 2.65^b^	0.24 ± 0.03^a^^b^	231.58 ± 21.37^a^^d^^e^^f^
8:16 HC	56.51 ± 2.34^b^	0.25 ± 0.01^b^	222.15 ± 13.41^a^^c^
12:12 HC	60.45 ± 1.38^d^	0.26 ± 0.01^b^	236.58 ± 12.63^b^^c^^e^
16:8 HC	54.55 ± 3.77^b^	0.22 ± 0.01^a^	251.72 ± 11.69^b^^f^

Data are the mean ± SD (*n* ≥ 3). Different letters represent significant difference (*p* < 0.05) among different treatments.

**Table 6:** Two-way ANOVA analysis of variance for the effects of CO_2_ and photoperiod regimes on maximum rETR (rETRmax), efficiency of electron transport (*α*), and saturating irradiance (I_k_) of *Ulva linza* cultured at different CO_2_ levels and photoperiod regimes.

Source	rETRmax			*α*			I_k_		
	df	F	Sig.	df	F	Sig.	df	F	Sig.
Photoperiod	2	2.981	0.066	2	2.909	0.070	2	7.030	0.003
CO_2_	1	69.993	<0.001	1	15.225	<0.001	1	0.664	0.421
Photoperiod* CO_2_	2	23.445	<0.001	2	14.896	<0.001	2	2.576	0.093
Error	30			30			30		

**Notes.**

CO_2_*photoperiod means the interactive effects of CO_2_ and photoperiod, df mean degree of freedom and F means the value of F statistic, and Sig. means *p*-value.

**Table 7:** Two-way ANOVA analysis of variance for the effects of CO_2_ and photoperiod regimes on respiration rate of *Ulva linza* cultured at different CO_2_ levels and photoperiod regimes.

Source	df	F	Sig.
Photoperiod	2	16.006	<0.001
CO_2_	1	33.058	<0.001
Photoperiod* CO_2_	2	9.566	0.003
Error	12		

**Notes.**

CO_2_*photoperiod means the interactive effects of CO_2_ and photoperiod, df mean degree of freedom and F means the value of F statistic, and Sig. means *p*-value.

**Table 8:** Two-way ANOVA analysis of variance for the effects of CO_2_ and photoperiod regimes on Chl *a*, Chl *b*, and Chl *a*/*b* of *Ulva linza* cultured at different CO_2_ levels and photoperiod regimes.

Source	Chl *a*			Chl *b*			Chl *a*/*b*		
	df	F	Sig.	df	F	Sig.	df	F	Sig.
Photoperiod	2	121.283	<0.001	2	111.035	<0.001	2	2.625	0.113
CO_2_	1	51.793	<0.001	1	53.849	<0.001	1	0.171	0.686
Photoperiod* CO_2_	2	26.932	<0.001	2	21.719	<0.001	2	8.389	0.005
Error	12			12			12		

**Notes.**

CO_2_*photoperiod means the interactive effects of CO_2_ and photoperiod, df mean degree of freedom and F means the value of F statistic, and Sig. means *p*-value.

## Discussion

In the present study, at LC, extended photoperiods had a positive effect on the relative growth rate of adult *U. linza,* similar to previous studies on *Laminaria sacharina, Porphyra umbilicalis* and *Ulva prolifera* (Fortes & Lüning, 1980; Green & Neefus, 2016; Li et al., 2018). Carbon isotope fractionation experiments suggested that extended photoperiods could enhance growth by influencing inorganic carbon capture and fixation rate in algae (Rost et al., 2003). This hypothesis is supported by the present study where extended photoperiods increased the maximum quantum yield in PS II. On the other hand, the highest growth rates of *Compsopogon coeruleus* were obtained in shorter light periods (L: D = 8:16) (Zucchi & Necchi, 2001), the highest growth rates of *Porphyra umbilicalis* was found under neutral (L: D = 12:12) and longer (L: D = 16:8) light periods using 110 µmol photons m^−2^s^−1^ (Green & Neefus, 2016), and the relative growth rate of *Chlorella vulgaris* cultured at L: D = 20:4 was lower than the RGR of alga cultured at L: D = 16:8 (Kendirlioglu, Agirman & Cetin, 2015). Therefore, the effects of photoperiod on algae appear to be species-specific.

Although *Ulva* has efficient mechanisms for CO_2_concentration, the growth of *Ulva* can be enhanced by elevated CO_2_ (Young & Gobler, 2016; Gao et al., 2016a; Gao et al., 2017a; Gao et al., 2017b). However, in this study, we found that the effects of elevated CO_2_ levels on the relative growth rate of *U. linza* depended on photoperiod. CO_2_ enhanced the growth of adult *U. linza* under light/dark conditions of 8:16, had no effect on growth of *U. linza* under a L: D of 12:12, and reduced the relative growth rate under a L: D of 16:8. High CO_2_ levels can down-regulate algal CO_2_ concentration mechanisms (CCMs), meaning that energy would be saved, and thus enhancing the relative growth rate of algae (Gao et al., 2012; Gao et al., 2016a; Raven, Beardall & Sánchez-Baracaldo, 2017). This is supported by decreased pigment synthesis in thalli at higher CO_2_ levels. However, higher CO_2_ levels did not affect the growth rate of *U. linza* at medium photoperiods. The neutral effects of CO_2_ on the growth of *U. rigida* (Rautenberger et al., 2015) and *U. linza* (Gao et al., 2018) were also reported. We speculate that this is a compromise between the positive effects of elevated CO_2_ and negative effects of decreased pH. The negative effect of decreased pH on growth was documented for the brown alga *Sargassum muticum* (Xu et al., 2017)*.* Algae might need to consume additional energy to act against acid–based perturbation caused by decreased pH, leading to reduced growth (Xu et al., 2017). This is supported by an enhanced respiration rate at the higher CO_2_ levels in this study. The phenomenon of an increased respiration rate of algae under elevated CO_2_ concentrations was found in *Hizikia fusiformis* (Zou, 2005), the microalgae *Phaeodactylum tricornutum* (Wu, Gao & Riebesell, 2010) and *Emiliania huxleyi* (Jin et al., 2015).

Furthermore, the higher CO_2_ level reduced the growth rate of *U. linza* under the longest photoperiod in the present study. This may be due to the combination of down-regulated CCMs and excess light energy. The operation of CCMs is an energy-consuming process and the down-regulation of CCMs can result in additional energy (Raven, Beardall & Sánchez-Baracaldo, 2017). Higher light can usually reduce algal photosynthetic activity (Singh & Singh, 2015; Gao et al., 2016a). The energy saved due to down-regulation of CCMs at HC combined with high light intensity could synergistically damage the algal photosystem and photosynthetic rate (Gao et al., 2012; Gao et al., 2016a). Although light intensity did not change among different CO_2_ treatments, the lengthened photoperiod may have similar effect to increased light intensity. This argument is supported by increased NPQ at longer photoperiods as NPQ is photo-protective process to dissipate excess light energy. Higher CO_2_ levels also reduced maximum quantum yield and stimulated the respiration rate of *U. linza* during the longest photoperiod in this study, leading to the decrease in growth. Enhanced respiration rate is a signal that organisms are fighting against stress and damage (Xu et al., 2017).

In addition to growth, the interactive effect of CO_2_ and photoperiod was also found in photosynthetic parameters. For instance, elevated CO_2_ increased Yield, rETRmax and *α* at shorter photoperiods (L: D of 8:16 and 12:12) but did not affect rETRmax and reduced Yield at longest photoperiod (L: D of 16:8). These findings indicate the close connection between growth and photosynthesis in terms of responding to the combination of CO_2_ and photoperiod. It is worth noting that the interaction of CO_2_ and photoperiod on growth of *U. linza* in this study is different from the findings in *U. prolifera* reported by Li et al. (2018). Elevated CO_2_ increased growth of *U. prolifera* at all three photoperiods (L: D of 12:12, 10:14 and 16:8). The different results may be due to differential physiological property between *U. linza* and *U. prolifera*. It has been documented that *U. prolifera* has a higher tolerance to high light intensity compared to *U. linza* (Cui et al., 2015). The strong capacity in dealing with high light intensity could contribute to the effect of elevated CO_2_ and prolonged photoperiod on growth *U. linza* was still positive. Integrating our findings with Li et al. (2018) study, the interaction of CO_2_ and photoperiod on *Ulva* species would be species dependent.

## Conclusions

This work is the first attempt to clarify the interaction between light/dark and elevated CO_2_ levels on the physiological responses of *Ulva linza*. We found that the effect of OA on *U. linza* depended on photoperiod. Outbreaks of green tides during spring and summer in China occur when the photoperiod is reaching its peak. Our findings indicate future OA may hinder the occurrence of green tides dominated by *U. linza* in combination with extended photoperiods. More environmental factors, such as temperature and nutrient levels, need to be investigated to obtain a more comprehensive understanding on development of green tides in future oceans.

##  Supplemental Information

10.7717/peerj.7048/supp-1 Data S1 Raw data Each data points indicates the average performance of triplicate.

## Additional Information and Declarations

### Field Study Permissions

The following information was supplied relating to field study approvals (i.e., approving body and any reference numbers): *U. linza* was collected from the coastal water of Gaogong peninsula (119.3°E, 34.5°N), Lianyungang, Jiangsu Province, China. Gaogong peninsula is a public place and no approval is required for collecting naturally growing Ulva species in China because Ulva species cause green tides in coastal waters of the Yellow Sea.

### Data Availability

The following information was supplied regarding data availability: The raw data are available in Data S1.
